# Surgical treatment for mediastinal abscess induced by endobronchial ultrasound-guided transbronchial needle aspiration: a case report and literature review

**DOI:** 10.1186/s12957-017-1206-4

**Published:** 2017-07-14

**Authors:** Yujiro Yokoyama, Takahiro Nakagomi, Daichi Shikata, Rumi Higuchi, Toshio Oyama, Taichiro Goto

**Affiliations:** 1 Department of General Thoracic Surgery, Yamanashi Central Hospital, Yamanashi, 400-8506 Japan; 2 Department of Pathology, Yamanashi Central Hospital, Yamanashi, Japan

**Keywords:** Endobronchial ultrasound-guided transbronchial needle aspiration (EBUS-TBNA), Mediastinitis, Lung cancer, Surgery

## Abstract

**Background:**

Endobronchial ultrasound-guided transbronchial needle aspiration (EBUS-TBNA) is a useful and less invasive procedure for the definitive diagnosis of mediastinal and hilar lymph nodes. However, infectious complications can occur after EBUS-TBNA, although they are extremely rare.

**Case presentation:**

A 66-year-old man with necrotic and swollen lower paratracheal lymph nodes underwent EBUS-TBNA. A mediastinal abscess developed 9 days post-procedure. Surgical drainage and debridement of the abscess were performed along with lymph node biopsy followed by daily washing of the thoracic cavity. Surgical treatment was effective, leading to remission of the abscess. Biopsy revealed that the tumor was squamous cell carcinoma with no radiologically detected cancer elsewhere in the body. Mediastinal lung cancer was thus confirmed. Subsequent chemoradiotherapy led to the remission of the tumor.

**Conclusions:**

Mediastinitis after EBUS-TBNA is rare but should be considered, particularly if the target lymph nodes are necrotic. Mediastinitis can lead to serious and rapid deterioration of the patient’s condition, for which surgical intervention is the treatment of choice.

## Background

Endobronchial ultrasound-guided transbronchial needle aspiration (EBUS-TBNA) is a minimally invasive method that is now being widely performed to obtain specimens from mediastinal and hilar lesions [1]. It is a useful tool not only for the staging of lung cancer but also for the diagnosis of diseases such as sarcoidosis and metastatic malignancy [2]. Although complications associated with EBUS-TBNA are rare [3], infectious complications have been reported [4–15]. We report a case of mediastinitis resulting from an infection after EBUS-TBNA which we successfully treated with prompt surgical drainage. Furthermore, we review the literature regarding infective complications of EBUS-TBNA and propose a possible mechanism of pathogenesis.

## Case presentation

**Fig. 1:**
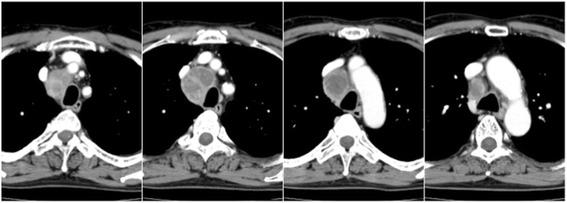
Chest CT before EBUS-TBNA showing swelling of the lower paratracheal lymph nodes. The lymph nodes are mostly necrotic

**Fig. 2:**
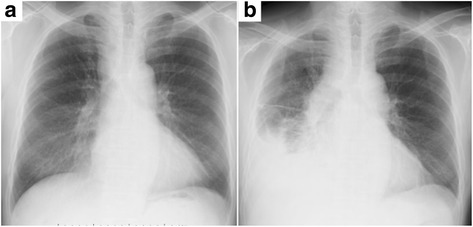
Chest radiographs after EBUS-TBNA. **a** Chest radiograph 1 day after EBUS-TBNA. **b** A chest radiograph 9 days after EBUS-TBNA showing enlargement of the mediastinum, increased pleural effusion, and an infiltrative shadow in the right lower lung field

A 66-year-old man was referred to our hospital for examination of swollen mediastinal lymph nodes. He was a current smoker with no significant past medical history. Computed tomography (CT) showed swelling of the lower paratracheal lymph nodes, with a maximum diameter of 48 mm; however, there were no pulmonary lesions (Fig. 1). EBUS-TBNA of the lymph nodes was performed with a total of 3 needle aspiration procedures under deep sedation by a well-trained bronchoscopist with 10 years’ experience in EBUS-TBNA. Prophylactic antibiotics were not administered, and there were no immediate complications. The patient was discharged without complications 1 day after the procedure (Fig. 2a). Histologic examination of the specimen detected only nonspecific inflammatory changes without any malignant or granulomatous findings.

**Fig. 3:**
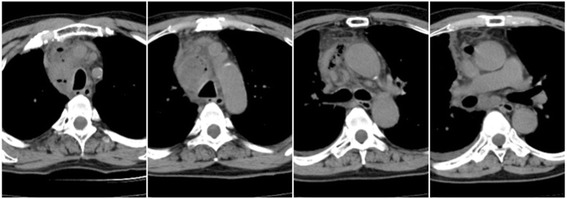
CT imaging of mediastinitis. Chest CT, performed 7 days after EBUS-TBNA, shows enlargement of the lower paratracheal lymph nodes and gas production in the mediastinum along with the appearance of right pleural effusion

Seven days after EBUS-TBNA, the patient experienced high fever and returned to our hospital. On admission, his symptoms included wheezing, right chest pain, and rapid breathing. The blood test results indicated infection: white cell count 9000/μL, C-reactive protein 33.58 mg/dL, and procalcitonin 50.76 ng/dL. The tumor markers, squamous cell carcinoma antigen (SCC) and cytokeratin-19 fragments (Cyfla), were elevated, being 5.2 and 3.9 ng/mL, respectively. Chest CT revealed right pleural effusion, enlargement of the lower paratracheal lymph nodes, and a multiloculated mediastinal abscess with gas production (Fig. 3). The patient was started on intravenous broad-spectrum antibiotics, which failed to improve his condition. A chest radiograph showed enlargement of the mediastinum, increased pleural effusion, and pneumonia in the right lower lung field (Fig. 2b); therefore, he was referred for surgical debridement and drainage along with lymph node dissection with curative intent. Nine days after EBUS-TBNA, surgery was performed via right posterolateral thoracotomy under general anesthesia. The intraoperative findings included fibrinopurulent fluid in the pleural cavity and mediastinum along with inflammatory swelling of the lymph nodes that rigidly adhered to the surrounding tissues. We decided that en bloc resection of the lymph nodes was not possible, so only a portion of these lymph nodes was biopsied. The thoracic cavity was washed with saline, and then a thoracic drain was inserted. Postoperatively, the patient remained critically ill with septic shock, and intrathoracic washing through the drain was started. Broad-spectrum antibiotics were continued as *Streptococcus pneumoniae* was isolated from multiple culture specimens of the debridement material. The patient’s general condition gradually improved, and he was discharged on antibiotics at 24 days post-procedure.

**Fig. 4:**
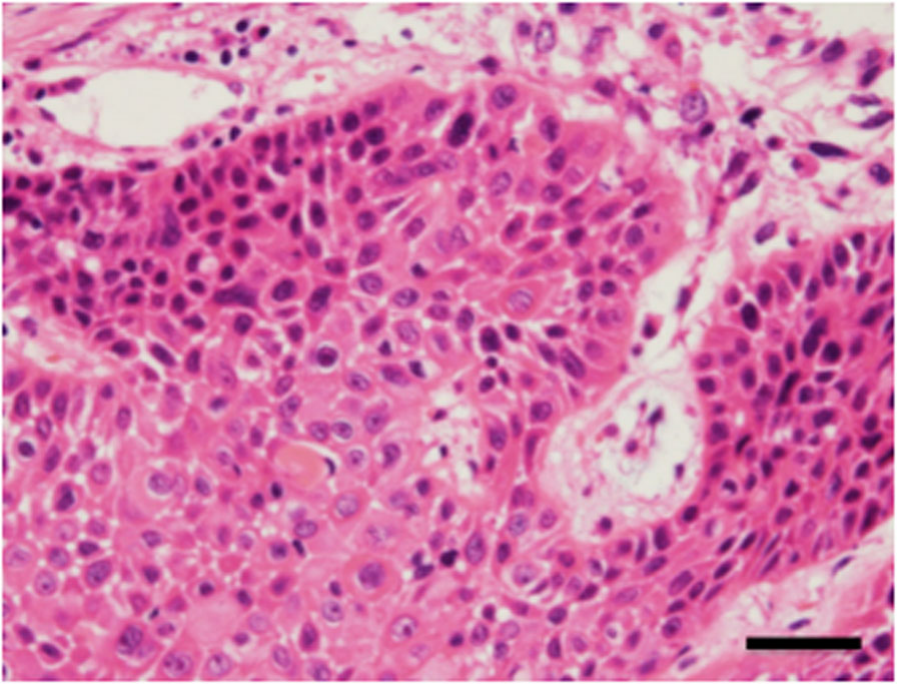
Histopathology of the biopsied specimen. The mediastinal lesion was diagnosed as squamous cell carcinoma. *Scale bar*, 40 μm

**Fig. 5:**
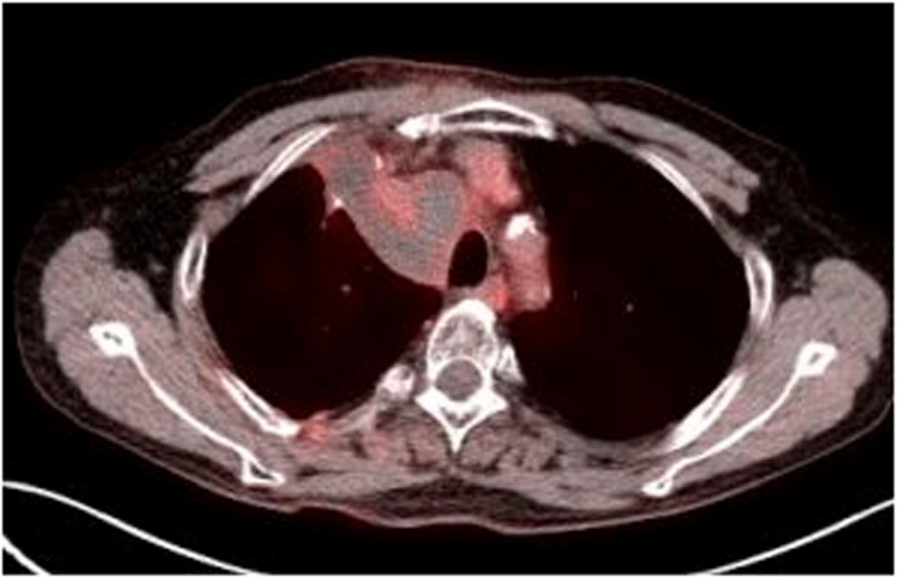
Positron emission tomography after chemoradiotherapy showing no fluorodeoxyglucose uptake in the mediastinal lesion

Pathological examination of the specimen revealed squamous cell carcinoma (Fig. 4), and the lesion was diagnosed as a mediastinal lung cancer because there were no primary cancer lesions elsewhere in the body. Thereafter, the patient received 6 courses of chemotherapy (carboplatin and nab-paclitaxel) with concurrent radiotherapy (70 Gy/35 Fr). The SCC and Cyfla levels returned to normal after chemoradiotherapy. Partial remission of the tumor was observed, and positron emission tomography showed no fluorodeoxyglucose uptake in these lesions (Fig. 5). The patient remains healthy without any progression of the disease 24 months after chemoradiotherapy.

## Discussion

EBUS-TBNA is a recently introduced, promising alternative to mediastinoscopy, which has been considered the standard technique in mediastinal lymph node biopsy [3, 16]. EBUS-TBNA has a high diagnostic yield with minimized invasiveness and reduced surgical complications [1, 3, 17–19]. Although a number of large-scale studies did not find complications associated with EBUS-TBNA [3], infectious complications have emerged as an issue with the increasing use of this procedure. In a Japanese survey, the frequency of infectious complications was reportedly 0.19% [20]. In particular, mediastinitis after EBUS-TBNA was reported in 0.10% of the cases [20].

To date, only 16 cases of infective mediastinitis after EBUS-TBNA have been reported (Table 1), but this apparently low rate of infection may be due to underreporting. The cases involved 14 men and 2 women with a mean age of 62.3 years (standard deviation (SD) = 14.2). Symptoms of mediastinitis occurred at a mean of 18.1 days (SD = 14.2) after EBUS-TBNA. In some cases, the symptoms manifested relatively late post-procedure, probably due to the small number of inoculating organisms. Clinical vigilance is necessary for an extended period after the intervention.

**Table 1:** Case list of mediastinitis caused by endobronchial ultrasound-guided transbronchial needle aspiration

Age	Sex	Lapsed days	Bacteria of cause	Diagnosis by EBUS	Surgical procedure	Ref.
73	M	11	N.P.	LN metastasis of lung cancer	N.P.	4
59	M	7	N.P.	LN metastasis of lung cancer	N.P.	5
68	M	60	*Streptococcus viridans*	LN metastasis of hepatocellular carcinoma	Thoracotomy	6
75	M	9	*Streptococcus intermedius*	Nonspecific inflammatory change	Median sternotomy	7
48	M	6	No bacteria	LN metastasis of lung cancer	Thoracotomy	8
68	M	35	*Candida albicans*, gamma-hemolytic *Streptococcus*	LN metastasis of colon cancer	EBUS-guided aspiration	9
66	M	14	*Propionibacterium acnes*, *Bacteroides*, *Eubacterium*	Undifferentiated, malignant cells	Thoracotomy	9
64	F	8	N.P.	LN metastasis of lung cancer	N.P.	10
72	M	10	Group C *Streptococcus*	LN metastasis of lung cancer	Thoracotomy	11
42	M	21	No bacteria	Sarcoidosis	Mediastinoscopy	12
89	F	14	Alpha-hemolytic *Streptococcus*, diphtheroids	Nonspecific inflammatory change	Thoracotomy	13
48	M	31	*Klebsiella pneumoniae*	Nonspecific inflammatory change	Thoracotomy	14
64	M	16	No bacteria	LN metastasis of lung cancer	Thoracotomy	15
49	M	14	*Gemella morbillorum*	Sarcoidosis	Mediastinotomy	15
36	M	26	*Prevotella buccae*, *Streptococcus anginosus*, *Actinomyces*	Sarcoidosis	Thoracotomy	15
75	M	7	*Streptococcus pneumoniae*	Mediastinal lung cancer	Thoracotomy	Our case

*F* female, *M* male, *N.P.* not performed, *LN* lymph node, *Ref.* reference

Surgery was performed in 12 of the 16 cases, and in 9 of these cases, thoracotomy was required. The most common treatment is thoracotomy with chest tube drainage and intravenous antibiotics [6, 8, 9, 11, 13–15]. In 4 cases, intravenous antibiotics alone were administered for 7 to 30 days and surgical debridement was unnecessary [4, 5, 9, 10]. The indication for the procedure was malignancy in 10 cases, particularly lymph node metastasis of lung cancer in 6 cases [4, 5, 8, 10, 11, 15]. In addition, the indications were sarcoidosis in 3 cases [12, 15] and nonspecific inflammatory changes in another 3 [7, 13, 14]. The implicated bacteria were of oropharyngeal origin, e.g., *Klebsiella pneumonia* [14], *Actinomyces* [15], hemolytic *Streptococcus* [9, 13], and *Streptococcus intermedius* [7]. No bacteria were detected in 3 cases, and the early administration of antibiotics eliminated the infectious pathogens. In all of the reported cases, mediastinitis resolved. The case we reported is the first instance of mediastinal lung cancer complicated by mediastinitis.

The presumed mechanism underlying the infectious complications is the introduction of oropharyngeal bacteria into deep mediastinal tissues during the transbronchial or transtracheal passage of the needle [21, 22]. In line with this theory of pathogenesis, *S. pneumoniae*, a common oropharynx contaminant, was identified as the culprit pathogen in the present case. In contrast, mediastinoscopy via a percutaneous approach is thought to be unlikely to cause infection with oropharyngeal bacteria. However, mediastinoscopy may be associated with other potentially catastrophic complications, such as bleeds due to major vessel damage, tracheobronchial injury, and esophageal trauma [16]. Several recent systematic reviews have reported the diagnostic accuracy of EBUS-TBNA is similar to mediastinoscopy with a significantly lower complication rate [23–25].

Matsuoka et al. suggested that biopsy needle puncture increases the risk of mediastinitis [11]. Infectious complications may be more likely after EBUS-TBNA on necrotic lesions because of compromised bacterial clearance. Based on previous reports of infectious complications after EBUS-TBNA, we suggest that in the case of necrotic lesions, EBUS-TBNA should be performed with great caution and prophylactic antibiotics need to be considered [8, 21, 26]. The antibiotics used in cases of clinically suspected infection must have activity against endogenous oral and nasopharyngeal organisms. Since there is no consensus on the use of prophylactic antibiotics for EBUS-TBNA at present, further investigation is needed.

Because mediastinitis after EBUS-TBNA is rare, the diagnosis is sometimes difficult. However, mediastinitis should be considered in the differential diagnosis because of its potentially fatal course. Mild mediastinitis is treatable with antibiotics alone, whereas surgical intervention should be considered without delay in more serious cases [4]. In our case, surgery was chosen immediately after the antibiotic treatment proved ineffective and severe mediastinitis was successfully treated with surgical drainage. In addition, mediastinal lung cancer was diagnosed using surgical biopsy, so that the patient was able to immediately start chemoradiotherapy. As a result, chemoradiotherapy, which is the gold standard of care in mediastinal lung cancer, was successful and the outcome favorable to date. This may be another valuable benefit of the performance of surgery.

## Conclusions

The present case underscores the risk of infectious complications after EBUS-TBNA. Close monitoring for the development of signs and symptoms of infection following EBUS-TBNA is essential. Surgery is the treatment of choice for an intractable mediastinal abscess, such as the case reported here.

## Abbreviations

CT: Computed tomography
Cyfla: Cytokeratin-19 fragments
EBUS-TBNA: Endobronchial ultrasound-guided transbronchial needle aspiration
SCC: Squamous cell carcinoma antigen
